# Evaluation of oral fluids for surveillance of foodborne and zoonotic pathogens in pig farms

**DOI:** 10.1177/10406387211021599

**Published:** 2021-06-02

**Authors:** Franziska Schott, Karolin Hoffmann, Eleonora Sarno, Patrick D. Bangerter, Roger Stephan, Gudrun Overesch, Michael Haessig, Xaver Sidler, Robert Graage

**Affiliations:** Department of Farm Animals, Division of Swine Medicine, University of Zurich, Zurich, Switzerland; Institute of Veterinary Pathology, University of Zurich, Zurich, Switzerland; Institute for Food Safety and Hygiene, University of Zurich, Zurich, Switzerland; Office for Consumer Protection Canton Aargau, Veterinary Service, Aarau, Switzerland (Bangerter); Institute for Food Safety and Hygiene, University of Zurich, Zurich, Switzerland; Institute of Veterinary Bacteriology, University of Bern, Bern, Switzerland; Administrative Department for Farm Animal Diagnostics, University of Zurich, Zurich, Switzerland; Department of Farm Animals, Division of Swine Medicine, University of Zurich, Zurich, Switzerland; Department of Farm Animals, Division of Swine Medicine, University of Zurich, Zurich, Switzerland

**Keywords:** hepatitis E virus, MRSA, pigs, saliva, *Salmonella* spp, Switzerland, *Yersinia enterocolitica*

## Abstract

The use of oral fluid (OF) to detect zoonotic pathogens in pigs has been only scarcely assessed. We evaluated OF as a potential specimen for detection by culture of methicillin-resistant *Staphylococcus aureus* (MRSA) and *Yersinia enterocolitica*, and the detection of antibodies against *Salmonella* spp. and hepatitis E virus (HEV) using commercial ELISAs. Samples from 33 pig farms were collected at the beginning and end of the fattening period. Results of the OF samples were compared with the results of serum samples and nasal swabs from individual pigs and pen floor fecal samples, using the Cohen kappa (κ) and the McNemar test. For *Salmonella* spp. antibodies, OF samples were negative, although the corresponding serum samples were positive. The detection of HEV antibodies in sera and OF had agreement at the first sampling, and poor and significant agreement at the second sampling (κ = 0.185, McNemar *p* = 0.238; κ = 0.088, McNemar *p* < 0.001). At both sampling times, the detection of MRSA in nasal swabs and OF showed agreement (κ = 0.466, McNemar *p* = 0.077; κ = 0.603, McNemar *p* = 1); agreement was seen for the detection of *Y.* *enterocolitica* in fecal and OF samples (κ = 0.012, McNemar *p* = 0.868; κ = 0.082, McNemar *p* = 0.061, respectively). According to the McNemar test, the use of pen-based OFs is more feasible for the detection of MRSA and *Y. enterocolitica* by culture than is detection of antibodies by commercial ELISA.

Pigs can act as asymptomatic carriers of various zoonotic pathogens. In particular, the consumption of raw or undercooked pork and pork products can result in the transmission to humans of pathogens such as *Salmonella* spp. and *Yersinia enterocolitica*.^4,39^ Hepatitis E is not only a disease transmitted via contaminated drinking water in developing countries with poor sanitary conditions, but also a zoonotic disease; domestic pigs, wild boars, and perhaps other animal species are reservoirs for hepatitis E virus (HEV; *Orthohepevirus A*). The occurrence of HEV in domestic pigs and wild boars raises concern for food safety.^12,26,34^ Pigs also act as a reservoir for methicillin-resistant *Staphylococcus aureus* (MRSA), and people with pig exposure are at higher risk for MRSA colonization or infection.^10,17,23^

Surveillance for foodborne and zoonotic pathogens might be beneficial to prevent the contamination of carcasses by asymptomatic carrier animals harboring such agents. Therefore, monitoring programs have been established to detect biological hazards at the herd level before slaughter. Conventional sampling methods in pigs, such as blood sampling and swabbing, take much effort, consume time and money, and cause considerable stress to the animals. Oral fluid (OF)-based testing offers an opportunity to gain pig herd health data at the farm in a simple and animal-friendly way.^30,33^ Pigs are naturally attracted to new and flexible objects^42^; in OF-based sampling, the pigs transfer OFs while chewing a rope.^19,27,32,37^

The immunoglobulin (Ig) fraction found in OF consists predominantly of IgA.^5,11^ Mucosal IgA antibodies are produced in plasma cells of local glandular tissue.^5^ IgG and IgM are also present in OF, although in lower quantities than IgA, and are derived primarily from plasma through ultrafiltration.^5,11^ Pathogen-specific IgA, IgM, and IgG antibodies have been demonstrated in OF collected from diverse domestic animal species in response to infection, making OF a useful matrix for immunologic assays.^30,31,33^ Also, various infectious agents are known to be shed by pigs in OF (e.g., foot-and-mouth disease virus, classical swine fever virus, porcine reproductive and respiratory syndrome virus [PRRSV]).^31^ OF testing is therefore considered an efficient and cost-effective approach for surveillance of viruses in swine herds.^19,33,35^ Nevertheless, little is known about the suitability of porcine OF for monitoring colonization by zoonotic bacteria, such as *Salmonella* spp., *Y. enterocolitica*, and MRSA, and infection by HEV.

We determined the feasibility of pen-based OF samples for generating data concerning carriage by pigs of potential zoonotic pathogens before slaughter. OF samples were taken twice; once when pigs were introduced on the fattening farm, and subsequently before the pigs were slaughtered. We investigated whether OF samples obtained under field conditions in pig herds on 33 fattening farms were suitable for the detection of antibodies against HEV and *Salmonella* spp. by commercial ELISAs. Given the possible colonization of the nasal cavity and oropharynx by MRSA and *Y. enterocolitica*, the detection of both was attempted by culture.^14,36^ We compared the results of OF herd sampling with the results obtained by nasal swabs for MRSA, or blood samples from individual animals for HEV and *Salmonella* spp., or pen floor fecal samples for *Yersinia.*

## Materials and methods

### Experimental design

Our study was conducted on 33 Swiss fattening farms, located in the area with the highest pig density in Switzerland, covering 9 Swiss cantons. Farms participated upon the request of the farm veterinarian (*n* = 12), pig trading companies (*n* = 10), the Swiss Pig Health Service (*n* = 2), or by the study director (*n* = 5). The farms were visited at the beginning and end of one fattening period (Table 1). Most of the farms (*n* = 20) were sampled either the day new fattening pigs arrived (day 0) or the next day (day 1). Eleven farms were sampled between days 2 and 4, and only 2 farms were sampled at another time because of farm management reasons. The newly arrived pigs weighed an average of 26 kg. Pigs were chosen haphazardly. In 23 farms, the same 1,409 ear-tagged animals were tested twice (beginning and end of fattening period) for the occurrence of *Salmonella* spp., *Y. enterocolitica*, and HEV using blood samples, nasal swabs, pen floor fecal samples, and OF samples (1 cotton rope per 20 pigs); 524 of these pigs were also tested for the occurrence of MRSA. In the 10 remaining farms, 294 pigs were tested twice (beginning and end of fattening period) only for the occurrence of MRSA using nasal swabs and OF specimens (Table 2). The second sampling took place 1 d to 3 wk before the pigs were slaughtered, at a weight of 80–100 kg and 5–6 mo old.

**Table 1.** Overview of farms participating in sampling of pigs for various pathogens.

Farm	No. of pigs sampled (1st/2nd sampling)	No. of pens sampled for OF (1st/2nd sampling)	Pig flow	Sampling times	Total no. of pigs on farm
1	70/70	6/6	All in, all out	2×	70
2	49/50	5/5	All in, all out	2×	50
3	76/76	6/12	Continuous	2×	88
4	55/55	2/3	Continuous	2×	63
5	10/9	1/1	Continuous	2×	10
6	40/38	1/1	Continuous	2×	40
7	87/82	3/4	All in, all out	2×	115
8	73/0	4/0	Continuous	1×	90
9	36/36	1/2	Continuous	2×	36
10	20/20	2/2	Continuous	2×	20
11	87/80	5/6	Continuous	2×	100–200
12	55/51	6/6	All in, all out	2×	51–100
13	101/97	8/7	All in, all out	2×	180
14	56/0	3/0	All in, all out	1×	94
15	92/79	5/7	Continuous	2×	125
16	68/67	2/2	Continuous	2×	80
17	25/18	1/3	Continuous	2×	30
18	119/119	27/27	All in, all out	2×	442
19	47/0	2/0	NA	1×	60
20	76/66	4/5	Continuous	2×	100
21	40/40	4/4	Continuous	2×	41
22	78/72	4/4	Continuous	2×	125
23	49/48	2/2	Continuous	2×	52
24	28/24	4/4	Continuous	2×	56
25	29/29	3/3	Continuous	2×	105
26	26/26	3/3	All in, all out	2×	201–300
27	39/39	1/1	Continuous	2×	1–50
28	25/25	1/6	Continuous	2×	1–50
29	30/30	1/2	Continuous	2×	51–100
30	27/21	2/2	NA	2×	NA
31	30/22	2/2	NA	2×	NA
32	30/27	1/2	Continuous	2×	51–100
33	30/29	1/2	Continuous	2×	51–100

NA = not assessed; OF = oral fluid.

**Table 2.** Overview of the test results of either oral fluid samples or the conventional sample matrices on pen level at the first and second sampling.

Pathogen	1st sampling (3-mo-old pigs)				2nd sampling (6-mo-old pigs)			
	Conventional sample matrices*		Oral fluid		Conventional sample matrices*		Oral fluid	
	Total pos. pens	Total pens tested	Total pos. pens	Total pens tested	Total pos. pens	Total pens tested	Total pos. pens	Total pens tested
*Salmonella* spp. (ELISA)	24 (23.1)	104	0 (0)	104	49 (45.4)	108	0 (0)	108
Hepatitis E virus (ELISA)	20 (19.2)	104	10 (9.6)	104	82 (75.9)	108	56 (51.9)	108
MRSA (culture)	11 (9.1)	121	5 (4.1)	121	8 (5.9)	136	9 (6.6)	136
*Yersinia enterocolitica* (culture)	31 (29.8)	104	27 (26.5)	102	26 (26.3)	99	12 (11.5)	104

Numbers in parentheses are percentages. Pos. = positive.

* Conventional sample matrices: detection of antibodies (IgG) against *Salmonella* spp. and hepatitis E virus in serum samples; culture of MRSA from nasal swabs; culture of *Yersinia enterocolitica* from pen floor fecal samples.

The sample size was based on the prevalence of each pathogen, the herd size, and the sensitivity and specificity of the tests used (Table 1).^7^ The reported seroprevalences for *Salmonella* spp. and HEV infections in finishing pigs in Switzerland are 4% and 60%, respectively.^41^ The prevalences of MRSA and *Y. enterocolitica* in Swiss fattening pigs were estimated to be ~20% and ~88%, respectively, therefore a smaller number of animals were sampled by nasal swabs.^2,15,28^ The farm visits were carried out according to the Swiss Animal Welfare guidelines (study LU 03/2014).

### Oral fluid sample collection

The OF collection procedure has been described elsewhere.^30,37^ In brief, OF samples were collected by hanging in each pen a 3-strand twisted, 12-mm diameter, unbleached cotton rope (Seilerei Kislig). If the pens contained >20 pigs, a second rope was placed. The ropes were positioned in the pens at the shoulder height of the pigs for at least 45 min, after which the rope was inserted into a single-use plastic bag and OF was extracted manually. The samples were transported to the Division of Swine Medicine (University of Zurich, Zurich, Switzerland); the OF was decanted into a 10-mL tube, and later pipetted into 1.8-mL cryotubes (Sarstedt). OF was shipped to 2 different laboratories (Institute for Food Safety and Hygiene, University of Zurich, Switzerland and Institute of Veterinary Bacteriology, University of Bern, Switzerland) and tested for antibodies against *Salmonella* spp. and HEV by ELISA, and for *Y. enterocolitica* and MRSA by culture. Samples for culture were stored overnight at 4°C and shipped the next day to the laboratory. Samples for ELISA were stored at −20°C until analyzed.

### Blood sample collection

Blood samples were centrifuged for 10 min at 1,000 × *g*, and sera were aliquoted into three 1.8-mL cryotubes (Sarstedt) and frozen at −20°C until analyzed. Sera were tested for antibodies against *Salmonella* spp. and HEV.

### Nasal swab sample collection

For MRSA detection, nasal swabs were taken from both nares from 815 pigs on 33 different fattening farms using transport swabs with Amies medium (Thermo Fisher). The swabs were stored overnight at 4°C and shipped to the laboratory the following day.

### Fecal sample collection

For *Y. enterocolitica* testing, fresh pooled fecal samples (5:1) were obtained from the floor of each pen in which sampled pigs were housed, placed in a clean 100-mL plastic tube, refrigerated at 4°C overnight, and shipped to the laboratory the following day.

### Laboratory analysis

Detection of *Y. enterocolitica* by culture, as well as HEV and *Salmonella* spp. antibodies by ELISA, was performed at the Institute for Food Safety and Hygiene, Vetsuisse Faculty, University of Zurich, and for MRSA by culture at the Institute of Veterinary Bacteriology, Vetsuisse Faculty, University of Bern.

Commercial serum ELISA kits (pigtype *Salmonella* Ab, Qiagen; PrioCHECK HEV antibody ELISA kit, Prionics) were used to detect IgG antibodies against *Salmonella* spp. and HEV, respectively, in porcine OF and sera. The detection of antibodies against HEV in OF was validated by using oral fluids spiked with HEV antibody–positive serum samples (data not shown). OF samples were assayed according to the manufacturers’ instructions for serum samples, with the exception that OFs were centrifuged first at 16,110 × g for 60 s and then pipetted onto a microwell plate without dilution. The following steps were conducted as indicated by the manufacturer.

For the detection of MRSA, 1 mL of OF was centrifuged for 10 min at 1,970 × g, and the supernatant was discarded. OF sediment and nasal swabs were transferred into tubes containing 10 mL of Mueller–Hinton broth supplemented with 6.5% NaCl. The following steps were performed as described previously.^28^ *S. aureus* was identified using matrix-assisted laser desorption/ionization time-of-flight mass spectroscopy (MALDI-TOF MS; Biotyper 3.0, MBT Compass Library DB-5989 MSP; Bruker Daltonics) following the direct transfer protocol recommended by the manufacturer. MRSA isolates were confirmed by PCR for the *mecA* gene as described previously.^38^

*Y. enterocolitica* was detected by mixing 1 mL of OF or 1 g of feces in 10 mL of phosphate-buffered saline (PBS) with 1% mannitol and 0.15% bile salts (PMB).^16,25^ After 2 wk of cold enrichment at 4°C, 10 μL of the enrichment was plated on cefsulodin–irgasan–novobiocin agar (Thermo Fisher), and the plates were incubated at 30°C for 24–48 h. Colonies with typical morphology were subcultured on blood agar and then tested for urease; this method is ISO-certified for identifying *Yersinia* spp.^21,22^

### Statistical analysis

Data recording and editing were performed (Excel 2007; Microsoft); statistical analyses were performed (Statistical Software: release 15.1; StataCorp). The results of conventional sampling methods were compared with the results from OF. A pen was assessed positive for a pathogen and antibodies if an individual serum sample or the pen-based OF sample was positive. Results from sera and OF were compared at the pen level. The sensitivity, specificity, positive and negative predictive values, and positive and negative likelihood ratios were assessed, setting serum samples, nasal swabs, or fecal samples as the gold standard. Given an ordinal distribution, the variables were not normally distributed. No statistical analysis or transformation for normal distribution was performed. The level of significance was set at 5%. Agreement of results obtained from OF and serum samples for ELISA, and OF from nasal swabs or fecal samples for culture, were determined using the McNemar test. The Cohen kappa (κ) was used to assess associations between positive ELISA and culture results and OF.^13,40^

## Results

**Figure 1.**
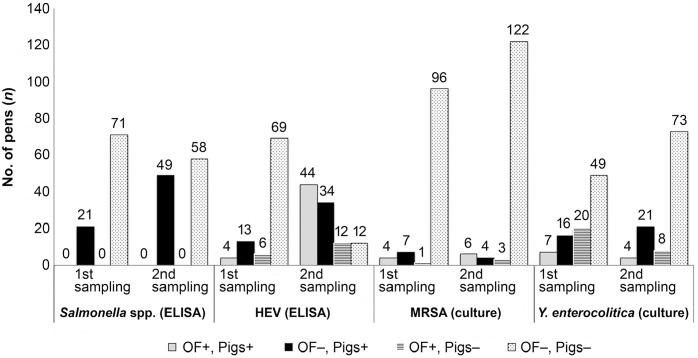
Number of pens that tested positive for antibodies to *Salmonella* spp. and hepatitis E virus (HEV) by ELISA, and positive for methicillin-resistant *Staphylococcus aureus* (MRSA) and *Yersinia enterocolitica* by culture: comparison of ELISA and culture results obtained on oral fluid (OF) with those obtained on other matrices (nasal swabs, sera, feces) from animals in the same herd. At the first sampling, 92 pens were tested for *Salmonella* spp. and HEV antibodies, and for *Y. enterocolitica*; 108 pens were tested for MRSA. At the second sampling, 107 pens were tested for *Salmonella* spp. and HEV antibodies, and for *Y. enterocolitica*; 135 pens were tested for MRSA. + = positive; − = negative.

From the initial 1,409 pigs, 1,173 pigs were retested at the second sampling for antibodies to *Salmonella* spp., HEV, and *Y. enterocolitica* (Fig. 1). An additional 294 pigs were tested for MRSA at the first sampling time, and 272 of these pigs were retested a second time (Fig. 1). In farms 8, 14, and 19, second sampling could not be performed.

In most pens, pigs interacted quickly with the rope provided, yet in several pens no OF was harvested. In farms 7 and 13, the same pigs would not chew the rope at either the first or the second sampling time. In farms 2, 5, 6, and 30, pigs would not chew on the rope the first day they arrived on the farm but chewed eagerly on the second day.

The apparent seroprevalence of *Salmonella* spp. in samples of individual animals was 0%–48.9% at the first sampling, and 0%–31.7% at the second sampling (Table 2, Suppl. Table 1). At both times, no *Salmonella* spp. IgG was detected in OF (Fig. 1, Table 2, Suppl. Table 1). The seroprevalence for HEV was up to 16.6% in serum samples and 83.3% in OF at the first sampling, and up to 97.9% in serum samples and 100% in OF at the second sampling (Table 2, Suppl. Table 1). The MRSA and *Y. enterocolitica* prevalence in nasal swabs or fecal samples and OF was up to 100% at the farm level at both times (Table 2, Suppl.Table 2).

Test performance measures of the different pathogens in OF samples compared to specimens sampled conventionally (serum, nasal swabs, and fecal samples) revealed variable results (Fig. 1, Table 3). For *Salmonella* spp. IgG, no positive predictive value and positive likelihood ratio could be calculated because no antibodies were detected for this pathogen in OF. The detection of specific IgG against HEV showed poor agreement between sera and OF at the first sampling (κ = 0.185, McNemar *p* = 0.238) and poor agreement at the second sampling (κ = 0.088, McNemar *p* < 0.001). The detection of MRSA showed moderate agreement between nasal swabs and OF at the first sampling (κ = 0.466, McNemar *p* = 0.077) and substantial agreement at the second sampling (κ = 0.604, McNemar *p* = 1). The detection of *Y. enterocolitica* showed poor agreement between fecal samples and OF at the first sampling (κ = 0.012, McNemar *p* = 0.868) and poor agreement at the second sampling (κ = 0.082, McNemar *p* = 0.061).

**Table 3.** Overview of the analyzed test performance measures of various pathogens in oral fluid samples compared to conventional samples (serum, nasal swabs, and fecal samples).

Pathogen	First sampling								Second sampling							
	SE	SP	PPV	NPV	LRp	LRn	κ	*p*	SE	SP	PPV	NPV	LRp	LRn	κ	*p*
IgG anti-*Salmonella* ELISA	0 (0)	1(0)	NA	0.772 (0.043)	NA	1 (0)	NA	NA	0 (0)	1 (0)	NA	0.558 (0.051)	NA	1 (0)	NA	NA
IgG anti-hepatitis E virus ELISA	0.250 (0.108)	0.921 (0.031)	0.400 (0.145)	0.854 (0.039)	3.167 (0.584)	0.814 (0.148)	0.185	0.238	0.543 (0.055)	0.522 (0.104)	0.800 (0.054)	0.245 (0.061)	1.136 (0.240)	0.876 (0.233)	0.088	<0.001
MRSA culture	0.364 (0.145)	0.990 (0.010)	0.800 (0.179)	0.932 (0.025)	35.273 (1.072)	0.643 (0.228)	0.466	0.077	0.600 (0.155)	0.974 (0.015)	0.667 (0.157)	0.966 (0.017)	23.400 (0.063)	0.411 (0.388)	0.604	1
*Y.e.* culture	0.292 (0.093)	0.710 (0.055)	0.259 (0.084)	0.742 (0.054)	1.006 (0.370)	0.997 (0.152)	0.012	0.868	0.208 (0.082)	0.885 (0.036)	0.357 (0.128)	0.784 (0.044)	1.806 (0.507)	0.895 (0.112)	0.082	0.061

Numbers in parentheses are standard errors. IgG = immunoglobulin G; κ = Cohen kappa; LRn = negative likelihood ratio; LRp = positive likelihood ratio; MRSA = methicillin-resistant *Staphylococcus aureus*; NA = not assessed; NPV = negative predictive value; *p*-value, McNemar test; PPV = positive predictive value; SE = sensitivity; SP = specificity; *Y.e.* = *Yersinia enterocolitica*.

## Discussion

OF is used as an alternative to serum for testing purposes, especially for monitoring, surveillance, and detection of pathogens and immunoglobulins at the herd level.^24,30,33^ OF offers a cost-effective approach because >80% of the pigs in the same pen are represented when presenting the rope for 30 to 45 min.^19,37^ Guidelines for OF sampling for the presence of PRRSV on farm demonstrate that using a fixed spatial sampling method, the probability of detecting PRRSV in one barn using 2 OF samples is 43% when the prevalence is 25%.^35^ In several studies, OF samples were considered to be useful for detecting zoonotic pathogens in pigs. OF is useful in detecting *Erysipelothrix* spp., *Streptococcus suis*, and influenza A virus in pigs.^3,8,9,18^

In some pens, no interaction with the rope was recorded, and no OF was harvested. The reason behind this unequal interest in the rope can only be guessed. In pens with fully slatted floors, increasing the number of ropes will usually lead to an increase in the total chewing-time in pigs, but no such effect is known in straw-bedded pens, which are customary in Switzerland.^37^ Therefore, we could not assess if straw bedding has an effect on the pig–rope interaction in our study. In some farms, pigs did not chew the rope at arrival. This behavior was probably caused by stress and distraction caused by many new impressions. If OF is used for monitoring and surveillance purposes, it is essential that pigs from all kinds of farms chew on the ropes provided. Alternatively, pigs could be sampled before they leave the farrowing site or some days after arrival at the fattening farm. Also, the time of day might be an influencing factor, given the fact that pigs are more active during the day. If a monitoring program for herd health prior to slaughter is established using OF, the collection should be done during the fattening period, but only after an adjustment period or at the end of the fattening period.

We evaluated the detection of anti-HEV and anti-*Salmonella* antibodies in OF specimens using commercial ELISAs that were not validated for OF. We modified the ELISA procedures only by the dilution of the OF samples for the detection of *Salmonella* spp. and HEV. Using a lower dilution (1:2) and overnight incubation of OF samples for detecting antibodies against *Salmonella* spp. might improve the performance of the ELISA.^1^ We did not incubate the sample overnight, and we sampled from naturally infected pigs with a mean seroprevalence of 4.4%. The low percentage of positive pigs in our study and the use of a non-validated ELISA might have influenced our results. However, further investigations are needed to provide reliable results in detecting antibodies against *Salmonella* spp. in OF by using an ELISA validated for serum. OF is feasible for the detection of antibodies against HEV in human saliva samples.^29^ Therefore, we tested negative OF spiked with a positive serum sample; HEV-specific antibodies were detectable (unpublished data). The validity of this approach was supported by prior evidence that pigs have detectable levels of antibodies in OF.^5,11^

Several hypotheses can be made for the negative outcomes in OF of the ELISAs for the detection of antibodies against *Salmonella* spp. One reason could be a lower diagnostic sensitivity of the method, given lower concentrations of IgG antibodies in OF compared to matched serum samples.^11,24^ Therefore, the ELISA for the detection of *Salmonella* spp. and HEV antibodies was performed without dilution. Additionally, the predominant antibody in OF is IgA, but the test kits used detect IgG antibodies. Given that OF is thought to be a filtrate of serum, IgG should be present in OF, but in lower concentrations.^5,11^ It is known that the sample material may affect the results of OF testing. For example, OF collected with cotton-based materials contains lower amounts of IgA compared to whole saliva samples in human OF, as well as in pig OF, but has no diminished effect on the amount of IgG.^27^ Furthermore, the ELISAs that we used are validated for serum and meat juice, not OF. OF is a different matrix, and, in addition to the lower antibody concentration, there might be inhibitory factors that reduce the amount of antibody in OF.^11,20^ If OF is stored, the amounts of detectable antibodies decrease over time.^32^

We used the Cohen kappa and the McNemar test to assess the agreement of conventional sample matrices and OF for the detection of HEV antibodies and the detection by culture of MRSA and *Y. enterocolitica*. Although the McNemar test failed to reject the null hypothesis of equal detection rates for the tested specimens, with the exception of the second sampling for HEV antibodies, the Cohen kappa showed only poor agreement of HEV antibodies or *Y. enterocolitica* culture between the corresponding samples. The reliability of the Cohen kappa for our study remains questionable in light of the high variability of farm-wise prevalence of positive pens as measured in the conventional sample matrices. Therefore, we considered the McNemar test more suitable for assessment of agreement between the conventional sample matrices and OF.

More pens tested positive for antibodies against HEV in serum samples than in OF. However, there was no significant disagreement between the 2 sample matrices. In farms 1 and 12, all pigs tested negative in serum for HEV, but OF tested positive. In several farms, positive sera were common, and OFs were either in agreement with sera or not. Further investigations are needed to provide feasible detection of antibodies against *Salmonella* spp. and HEV. The discrepancy of the ELISA results might be the result of cross-reaction with antibodies against different pathogens or in processing of OF. For example, ELISAs for detection of antibodies against PRRSV or *Erysipelothrix* spp. in OF seem to give good results after modification of the assay.^3,18,24^ It might be that the discrepancy of the results from the ELISAs used is the result of differences in the immune response generated by the pathogens (HEV, *Salmonella* spp.) tested.

A parallel study including this set of samples showed that antibodies against *Toxoplasma gondii* could be detected in OF from infected pigs by immunoblot (IB) techniques, and that IgA seemed to be a more adequate target than IgG.^6^ In that study, positive IB results were obtained in pooled OF samples from groups with high rates (>90%) of pigs seropositive to *T. gondii*, but not in groups with ≤13% seropositive pigs, suggesting that this approach might be used as a screening tool to determine high exposure to *T. gondii* in a farm.^6^ We showed that the use of OF as a screening tool for pig herd health for the tested pathogens using commercial ELISA kits, which are not adapted to the matrix of OF, is not promising at this point. Therefore, further research is needed into the use of ELISAs and in the detection of IgG against various pathogens in OF. It will be important to sample individual animals or all animals in a pen to ascertain the percentage of positive animals needed for pen-based OF samples to test positive.

We cultured OF in an effort to detect MRSA and *Y. enterocolitica*.^22,28^ This approach was valid given that both pathogens might be colonizing the oral cavity. We suspected that these 2 pathogens would also be present in OF given the proximity of the colonized organs. Only for the detection of MRSA in OF and nasal swabs from individual pigs by culture was substantial agreement found between the test results of the different specimens. MRSA are easily transmitted between animals and intermittently colonize the nasal cavity. Therefore, the MRSA status might change during the lifespan of a pig.^2^ MRSA are natural occupants of mucous membranes and the skin of the pig and might be more easily transmitted via the oral cavity. *Y. enterocolitica* persists deep in the tonsils and is intermittently transmitted via feces and might be transmitted in oral secretions.^36^ Interestingly, the percentage of detected *Y. enterocolitica* was higher in OF than in fecal samples at the second sampling. However, the McNemar test demonstrated agreement between OF and the reference matrices. The Cohen kappa and McNemar test showed different levels of agreement, given the high variance of positive pens in our study. The results for *Y. enterocolitica* cultural testing via OF might be beneficial. However, further investigation is needed to determine if OF is a beneficial matrix for the detection of *Y. enterocolitica.*

## Supplemental Material

sj-pdf-1-vdi-10.1177_10406387211021599 – Supplemental material for Evaluation of oral fluids for surveillance of foodborne and zoonotic pathogens in pig farms Click here for additional data file. Supplemental material, sj-pdf-1-vdi-10.1177_10406387211021599 for Evaluation of oral fluids for surveillance of foodborne and zoonotic pathogens in pig farms by Franziska Schott, Karolin Hoffmann, Eleonora Sarno, Patrick D. Bangerter, Roger Stephan, Gudrun Overesch, Michael Haessig, Xaver Sidler and Robert Graage in Journal of Veterinary Diagnostic Investigation
