# Supplementary material for: Evaluation of oral fluids for surveillance of foodborne and zoonotic pathogens in pig farms
Source: J Vet Diagn Invest. 2021 Jun 2;33(4):655–63. doi: 10.1177/10406387211021599 (PMC8225685; doi:10.1177/10406387211021599)
Supplement: sj-pdf-1-vdi-10.1177_10406387211021599 – Supplemental material for Evaluation of oral fluids for surveillance of foodborne and zoonotic pathogens in pig farms [file sj-pdf-1-vdi-10.1177_10406387211021599.pdf]

Schott F, et al. Evaluation of oral fluids for surveillance of foodborne and zoonotic pathogens in pig farms

Supplementary Table 1. Samples tested by ELISA for antibodies against *Salmonella* and hepatitis E virus (HEV).

	1st sampling (3-mo-old pigs)				2nd sampling (6-mo-old pigs)			
	IgG anti-*Salmonella*		IgG anti-HEV		IgG anti-*Salmonella*		IgG anti-HEV	
	Serum	Oral fluid	Serum	Oral fluid	Serum	Oral fluid	Serum	Oral fluid
Farm	Pos. pigs/no. pigs<br>No. pos. pens/no. pens	Pos. pens/no. pens	Pos. pigs/no. pigs<br>No. pos. pens/no. pens	Pos. pens/no. pens	Pos. pigs/no. pigs<br>No. pos. pens/no. pens	Pos. pens/no. pens	Pos. pigs/no. pigs<br>No. pos. pens/no. pens	Pos. pens/no. pens
1	**6**/70 (8.6)<br>**3**/6 (50)	0/6 (0)	0/70 (0)<br>0/6 (0)	**5**/6 (83.3)	**13**/70 (18.6)<br>**5**/6 (83.3)	0/6 (0)	0/70 (0)<br>0/6 (0)	**6**/6 (100)
2	0/49 (0)<br>0/5 (0)	0/5 (0)	**2**/49 (4)<br>**2**/5 (40)	0/5 (0)	**3**/50 (6)<br>**3**/5 (60)	0/5 (0)	**45**/50 (90)<br>**5**/5 (100)	0/5 (0)
3	**1**/76 (1.3)<br>**1**/6 (16.7)	0/6 (0)	**10**/76 (12.6)<br>**4**/6 (66.7)	**2**/6 (33.3)	**1**/76 (1.3)<br>**1**/12 (8.3)	0/12 (0)	**38**/76 (50)<br>**9**/12 (75)	0/12 (0)
4	**1**/55 (1.8)<br>**1**/2 (50)	0/2 (0)	**2**/55 (3.6)<br>**2**/2 (100)	**2**/2 (100)	**2**/55 (3.6)<br>**1**/3 (33.3)	0/3 (0)	**31**/55 (56.4)<br>**3**/3 (100)	0/3 (0)
5	0/10 (0)<br>0/1 (0)	0/1 (0)	**1**/10 (10)<br>**1**/1 (100)	0/1 (0)	0/9 (0)<br>0/1 (0)	0/1 (0)	0/9 (0)<br>0/1 (0)	0/1 (0)
6	0/40 (0)<br>0/1 (0)	0/1 (0)	**1**/40 (2.5)<br>**1**/1 (100)	**1**/1 (100)	0/38 (0)<br>0/1 (0)	NA	**28**/38 (73.7)<br>**1**/1 (100)	NA
7	**4**/87 (4.5)<br>**3**/3 (100)	0/3 (0)	0/87 (0)<br>0/3 (0)	0/3 (0)	**26**/82 (31.7)<br>**4**/4 (100)	0/4 (0)	**10**/82 (12.2)<br>**3**/4 (75)	0/4 (0)
8	0/73 (0)<br>0/4 (0)	0/4 (0)	**1**/73 (1.3)<br>**1**/4 (25)	0/4 (0)	NA	NA	NA	NA
9	**3**/36 (8.3)<br>**1**/1 (100)	0/1 (0)	**6**/36 (16.6)<br>**1**/1 (100)	0/1 (0)	7/36 (19.4)<br>**2**/2 (100)	0/2 (0)	**21**/36 (58.3)<br>**2**/2 (100)	0/2 (0)
10	0/20 (0)<br>0/2 (0)	0/2 (0)	0/20 (0)<br>0/2 (0)	0/2 (0)	**1**/20 (5)<br>**1**/2 (50)	0/2 (0)	0/20 (0)<br>0/2 (0)	0/2 (0)
11	0/87 (0)<br>0/5 (0)	0/5 (0)	0/87 (0)<br>0/5 (0)	0/5 (0)	0/80 (0)<br>0/6 (0)	0/6 (0)	**65**/80 (81.3)<br>**6**/6 (100)	0/6 (0)
12	0/55 (0)<br>0/6 (0)	0/6 (0)	0/55 (0)<br>0/6 (0)	0/6 (0)	**1**/51 (2)<br>**1**/6 (16.7)	0/6 (0)	0/51 (0)<br>0/6 (0)	**6**/6 (100)

13	0/101 (0)<br>0/8 (0)	0/8 (0)	0/101 (0)<br>0/8 (0)	0/8 (0)	**23**/97 (23.7)<br>**7**/7 (100)	0/7 (0)	**54**/97 (55.7)<br>**7**/7 (100)	**2**/7 (28.6)
14	**7**/56 (12.5)<br>**3**/3 (100)	0/3 (0)	0/56 (0)<br>0/3 (0)	0/3 (0)	NA	NA	NA	NA
15	**1**/92 (1.1)<br>**1**/5 (20)	0/5 (0)	0/92 (0)<br>0/5 (0)	0/5 (0)	**7**/79 (8.9)<br>**4**/7 (57.1)	0/7 (0)	0/79 (0)<br>0/7 (0)	0/7 (0)
16	**3**/68 (4.4)<br>**2**/2 (100)	0/2 (0)	**10**/68 (14.7)<br>**2**/2 (100)	0/2 (0)	**12**/67 (17.9)<br>**2**/ 2 (100)	0/2 (0)	**38**/67 (56.7)<br>**2**/2 (100)	0/2 (0)
17	**2**/25 (8.0)<br>**1**/1 (100)	0/1 (0)	0/25 (0)<br>0/1 (0)	0/1 (0)	**5**/18 (27.8)<br>**3**/3 (100)	0/3 (0)	**16**/18 (88.9)<br>**3**/3 (100)	**2**/3 (66.7)
18°	**5**/119 (4.2)<br>**4**/27 (14.8)	0/27 (0)	**7**/119 (5.8)<br>**5**/27 (18.5)	0/27 (0)	**3**/119 (2.5)<br>**3**/27 (11.1)	0/27 (0)	**92**/119 (77.3)<br>**26**/27 (96.2)	**25**/27 (92.6)
19	**23**/47 (48.9)<br>**2**/2 (100)	0/2 (0)	**2**/47 (4.2)<br>**1**/2 (50)	0/2 (0)	NA	NA	NA	NA
20	**3**/76 (3.9)<br>**2**/4 (50)	0/4 (0)	0/76 (0)<br>0/4 (0)	0/4 (0)	**18**/66 (27.3)<br>**5**/5 (100)	0/5 (0)	**64**/66 (97.0)<br>**5**/5 (100)	**5**/5 (100)
21	0/40 (0)<br>0/4 (0)	0/4 (0)	0/40 (0)<br>0/4 (0)	0/4 (0)	**1**/40 (2.5)<br>**1**/4 (25)	0/4 (0)	**25**/40 (62.5)<br>**4**/4 (100)	**4**/4 (100)
22	0/78(0)<br>0/4 (0)	0/4 (0)	0/78 (0)<br>0/4 (0)	0/4 (0)	**8**/72 (11.1)<br>**4**/4 (100)	0/4 (0)	**66**/72 (91.7)<br>**4**/4 (100)	**4**/4 (100)
23	0/49 (0)<br>0/2 (0)	0/2 (0)	0/49 (0)<br>0/2 (0)	0/2 (0)	**9**/48 (18.8)<br>**2**/ 2 (100)	0/2 (0)	**47**/48 (97.9)<br>**2**/ 2 (100)	**2**/2 (100)

NA = not assessed. Positive samples are marked in boldface; prevalence (%) is in parentheses.

Supplementary Table 2. Samples from pigs at 3 and 6 mo old tested by culture for methicillin-resistant *Staphylococcus aureus* (MRSA) and *Yersinia enterocolitica*.

	1st sampling (3-mo-old pigs)				2nd sampling (6-mo-old pigs)			
	MRSA		*Yersinia enterocolitica*		MRSA		*Yersinia enterocolitica*	
	Nasal swab	Oral fluid	Pen-based fecal samples	Oral fluid	Nasal swab	Oral fluid	Pen floor fecal samples	Oral fluid
Farm	No. pos. pigs/no. pigs<br>No. pos. pens/no. pens	No. pos. pens/no. pens	No. pos. pens/no. pens	No. pos. pens/no. pens	No. pos. pigs/no. pigs<br>No. pos. pens/no. pens	No. pos. pens/no. pens	No. pos pens/no. pens	No. pos. pens/no. pens
1	**7**/35 (20)<br>**4**/6 (67)	0/6 (0)	**2**/6 (33.3)	**1**/6 (16.7)	0/35 (0)<br>0/6 (0)	0/6 (0)	**6**/6 (100)	**0**/6 (0)
2	0/25 (0)<br>0/5 (0)	0/5 (0)	**2**/5 (40)	**1**/5 (20)	0/26 (0)<br>0/5 (0)	0/5 (0)	**1**/5 (20)	**2**/5 (40)
3	0/27 (0)<br>0/6 (0)	0/6 (0)	0/6 (0)	0/6 (0)	0/25 (0)<br>0/12 (0)	0/12 (0)	0/12 (0)	0/12 (0)
4	0/28 (0)<br>0/2 (0)	0/2 (0)	0/2 (0)	0/2 (0)	0/28 (0)<br>0/3 (0)	**1**/3 (0)	**3**/3 (100)	**1**/3 (33.3)
5	0/10 (0)<br>0/1 (0)	0/1 (0)	0/1 (0)	0/1 (0)	0/9 (0)<br>0/1 (0)	0/1 (0)	0/1 (0)	0/1 (0)
6	**13**/21 (62)<br>**1**/1 (100)	**1**/1 (100)	0/1 (0)	0/1 (0)	**19**/20 (95)<br>**1**/1 (100)	**1**/1 (100)	**1**/1 (100)	**1**/1 (100)
7	0/30 (0)<br>0/3 (0)	0/3 (0)	**2**/3 (66.7)	0/3 (0)	**1**/28 (4)<br>**1**/4 (25)	0/4 (0)	**2**/4 (50)	**4**/4 (100)
8	**1**/25 (4)<br>**1**/4 (25)	0/4 (0)	**1**/4 (25)	0/4 (0)	NA	NA	NA	NA
9	NA	NA	0/1 (0)	0/1 (0)	**21**/22 (96)<br>**2**/2 (100)	**2**/2 (100)	**1**/2 (50)	**1**/2 (50)
10	**20**/20 (100)<br>**2**/2 (100)	**2**/2 (100)	0/2 (0)	**2**/2 (100)	**20**/20 (100)<br>**2**/2 (100)	**2**/2 (100)	NA	0/2 (0)
11	0/29 (0)<br>0/5 (0)	0/5 (0)	0/5 (0)	**2**/5 (40)	0/26 (0)<br>0/6 (0)	0/6 (0)	0/6 (0)	**1**/6 (16.7)
12	0/28 (0)<br>0/6 (0)	0/6 (0)	**1**/6 (16.7)	**2**/6 (33.3)	0/27 (0)<br>0/6 (0)	0/6 (0)	**2**/6 (33.3)	0/6 (0)
13	0/27 (0)<br>0/8 (0)	**1**/8 (25)	**7**/8 (87.5)	**1**/8 (25)	0/29 (0)<br>0/7 (0)	**2**/7 (28.6)	**2**/7 (28.6)	0/7 (0)
14	0/19 (0)<br>0/3 (0)	0/3 (0)	**1**/3 (33.3)	**2**/3 (66.7)	NA	NA	NA	NA
15	0/27 (0)<br>0/5 (0)	0/5 (0)	**1**/5 (20)	**2**/5 (40)	0/21 (0)<br>0/7 (0)	0/7 (0)	**2**/7 (28.6)	**1**/7 (14.3)
16	0/24 (0)<br>0/2 (0)	0/2 (0)	**1**/2 (50)	**2**/2 (100)	0/24 (0)<br>0/2 (0)	0/2 (0)	0/2 (0)	0/2 (0)

17	0/17 (0)<br>0/1 (0)	0/1 (0)	0/1 (0)	**1**/1 (100)	0/11 (0)<br>0/3 (0)	0/3 (0)	0/3 (0)	0/3 (0)
18°	0/40 (0)<br>0/27 (0)	0/27 (0)	**3**/27 (11.1)	**9**/27 (33.3)	0/40 (0)<br>0/27 (0)	0/27 (0)	**6**/27 (22.2)	**1**/27 (3.7)
19	0/21 (0)<br>0/2 (0)	0/2 (0)	**2**/2 (100)	NA	NA	NA	NA	NA
20	**1**/25 (4)<br>**1**/4 (25)	0/4 (0)	0/4 (0)	0/4 (0)	0/23 (0)<br>0/5 (0)	0/5 (0)	0/5 (0)	0/5 (0)
21	0/20 (0)<br>0/4 (0)	0/4 (0)	**3**/4 (75)	0/4 (0)	0/21 (0)<br>0/4 (0)	0/4 (0)	0/4 (0)	0/4 (0)
22	0/26 (0)<br>0/4 (0)	0/4 (0)	**4**/4 (100)	**2**/4 (50)	0/25 (0)<br>0/4 (0)	0/4 (0)	0/4 (0)	0/4 (0)
23	NA	NA	**1**/2 (50)	0/2 (0)	0/24 (0)<br>0/4 (0)	0/4 (0)	0/4 (0)	0/4 (0)
24	0/28 (0)<br>0/4 (0)	0/4 (0)	NA	NA	0/24 (0)<br>0/4 (0)	0/4 (0)	NA	NA
25	0/29 (0)<br>0/3 (0)	0/3 (0)	NA	NA	0/29 (0)<br>0/3 (0)	0/3 (0)	NA	NA
26	0/26 (0)<br>0/3 (0)	0/3 (0)	NA	NA	0/26 (0)<br>0/3 (0)	0/3 (0)	NA	NA
27	0/39 (0)<br>0/1 (0)	0/1 (0)	NA	NA	0/39 (0)<br>0/1 (0)	0/1 (0)	NA	NA
28	0/25 (0)<br>0/1 (0)	0/1 (0)	NA	NA	0/25 (0)<br>0/6 (0)	0/6 (0)	NA	NA
29	0/30 (0)<br>0/1 (0)	0/1 (0)	NA	NA	0/30 (0)<br>0/2 (0)	0/2 (0)	NA	NA
30	0/27 (0)<br>0/2 (0)	0/2 (0)	NA	NA	0/21 (0)<br>0/2 (0)	0/2 (0)	NA	NA
31	0/30 (0)<br>0/2 (0)	0/2 (0)	NA	NA	0/22 (0)<br>0/2 (0)	0/2 (0)	NA	NA
32	**29**/30 (97)<br>**1**/1 (100)	0/1 (0)	NA	NA	**27**/27 (100)<br>**1**/2 (50)	0/2 (0)	NA	NA
33	**30**/30 (100)<br>**1**/1 (100)	**1**/1 (100)	NA	NA	**21**/29 (72)<br>**1**/2 (50)	**1**/2 (50)	NA	NA

NA = not assessed. Positive samples are marked in boldface; prevalence (%) is in parentheses.
